# Prevalence of G6PD deficiency in Children with Hepatitis A

**Published:** 2017-04-01

**Authors:** Ghasem Miri-Aliabad, Ali Khajeh, Tooran Shahraki

**Affiliations:** 1 Assistant Professor of Pediatric Hematology-Oncology, Children and Adolescent Health Research Center, Zahedan University of Medical Sciences, Zahedan, Iran; 2 Department of Pediatrics, Children and Adolescent Health Research Center, Zahedan University of Medical Sciences, Zahedan, Iran

**Keywords:** Children, Hepatitis A, G6PD deficiency

## Abstract

**Introduction** **: **Hepatitis A virus is the most prevalent viral hepatitis. It is globally a major public health problem with different clinical symptoms. This study aimed at investigating the clinical findings and prevalence of glucose 6-phosphate dehydrogenase (G6PD) deficiency in children with hepatitis A.

**Materials and Methods** **: **In this prospective study, demographical information, clinical findings, and G6PD level of hepatitis A patients, who were visited at Pediatric Hematology clinic, were entered into the database. The diagnosis of hepatitis A infection was based on the presence of anti-HAV IgM antibody. The activity of G6PD enzyme was measured with florescent spot test.

**Results** **: **Of the 117 children with hepatitis A, 52 (44.4%) were male and 65 (55.6%) were female. The mean age of these patients was 2.79±5.39 years. The most prevalent clinical manifestations were dark yellow urine and anorexia. G6PD deficiency was observed in 26 (26.3%) out of 99 patients whose G6PD levels were measured.

**Conclusion** **: **Given the high prevalence of G6PD deficiency in this study, the measurement of G6PD level along with other liver and biochemical markers in areas with endemic hepatitis A is recommended. In addition, it is recommended that patients undertake precise monitoring for hemolysis and renal function.

## Introduction

 The hepatitis A virus is a RNA virus belonging to the Picornaviridae family.^1^The virus usually spreads via the fecal-oral route, principally through ingestion of contaminated water, food, or by direct contact with an infected person. It is the most common cause of acute hepatitis in the world.^2^The prevalence of hepatitis A is linked with public socioeconomic and health status. Meanwhile, its incidence is less expected in developed countries.^3^^, ^^4^ Hepatitis A infection in children is usually asymptomatic or associated with mild symptoms, whereas it manifests with more severe symptoms in adults.^5^Extrahepatic manifestations are not unusual in hepatitis A infection.^6^ One of the most prevalent types of extrahepatic manifestations in these patients is hematologic disorders.^7^ Bone marrow hypoplasia has been reported in some cases with viral hepatitis.^8^ A study on patients with acute viral hepatitis showed that about 24% of patients had anemia, 16% had disorder in white cells count, 4% had leukopenia, 12% had leukocytosis, and about one third of patients had coagulation disorders, whereas none of them was diagnosed with thrombocytopenia.^7^ Hematocrit gradually decreases in the first three weeks after the onset of acute viral hepatitis due to the transient suppression of bone marrow and viral hepatitis-induced immune hemolytic process.^7^^,^^9^ G6PD deficiency is the most common enzyme deficiency in the world. Its prevalence ranges from 1% to 24% in various regions in Iran. Different factors can lead to incidence of hemolysis in patients with such deficiency.^10^This study aimed at investigating the demographical information, clinical symptoms, and the prevalence of G6PD deficiency in children with hepatitis A. 

## SUBJECTS AND METHODS

 This cross-sectional descriptive study was done on 117 patients (aged <18 years) with acute viral hepatitis in Ali-Asghar Hospital. In this prospective study, demographic information, clinical findings, and G6PD level of hepatitis A patients, who were visited at Pediatric clinic, were entered into the database. Demographic information of the patients including age, gender, history of contact with peers, findings from patients' clinical history and physical examination such as anorexia, abdominal pain, fever, nausea, vomiting, discolored urine, jaundice, hepatomegaly, and right upper quadrant tenderness was entered into the database. In addition, patients were examined for normal or deficient levels of G6PD. The diagnosis of hepatitis A infection was based on the presence of anti-HAV Ig M antibody. The activity of G6PD enzyme was measured with fluorescent spot test. All statistical data analyses were performed using SPSS version 22. The quantitative variables were shown as mean ± SD. The Chi-Square Test was used to compare qualitative variables and p-value<0.05 was considered as statistically significant.

## Results

 The demographic and clinical characteristics of the patients are presented in Table 1. In this study, 117 children (within the age range of 1.5 -13 years) were examined. The mean age of patients (n=117) including 65 (55%) males and 52 (44.4%) females was 5.39±2.79 years.

The most common clinical manifestation in these children was dark-yellow urine. At the time of admission, 89 (76.1%), 5 (5.1%), and 22 (18.8%) patients received healthcare and health education only at home, at kindergarten and home, and in school and at home, respectively. There was a history of contact with another child diagnosed with hepatitis A during the past 2-4 weeks in 28 (23.9%) subjects. Hepatomegaly and RUQ tenderness were seen in 40 (34.2%) and 70 (59.8%) participants, respectively. G6PD levels were measured in 99 (84.6%) of 117 patients. Results showed G6PD deficiency in 26 (26.3%) and normal G6PD level in 73 (73.7%) patients. 

Of 26 children with G6PD deficiency, 8 (31%) were female and 18 (69%) were male. The mean hemoglobin levels were 11.3±1.8 gr/dl and 10.8±2.2 gr/dl in children with normal and G6PD deficiency, respectively. The mean hemoglobin levels were 11.8±2.1 gr/dl and 11.1±1.5 gr/dl in male and female patients with normal G6PD level, respectively (p=0.12). The mean hemoglobin level was 10.5±1.9 gr/dl and 11.6±2.9 gr/dl in male and female patients with G6PD deficiency, respectively (p=0.25). 

**Table 1:** Demographic and clinical characteristics of the patients

**Parameter**	**No.**	**Percentage**
**Male gender**	52	44.4
**Icterus**	107	91.5
**Anorexia**	111	94.9
**Abdominal** **pain**	95	81.2
**Dark yellow** **urine**	115	98.3
**Weakness**	99	84.6
**Vomiting**	42	35.9
**Nausea**	67	57.3
**Fever**	77	65.8

## Discussion

 In this study, we investigated the prevalence of G6PD deficiency and clinical symptoms in children with hepatitis A in an outpatient clinic. The prevalence of G6PD deficiency in this study was 26.3%, however, it was reported to be 7% in a cross-sectional descriptive study on 1440 healthy subjects, of whom 84% had Mediterranean mutation.^10^ In a study of 280 schoolboys in Yasouj, 12.5% of whom had G6PD deficiency,^11^ which resulted in the most common inherited enzyme defects so far described.^12^ This enzyme deficiency has affected more than 400 million people worldwide.^13^ Infections or exposure to certain drugs and chemicals may lead to hemolytic attack and severe anemia in such patients. Infections such as viral hepatitis, typhoid fever, and pneumonia, as well as upper respiratory and gastrointestinal infections are well known to be the triggers of hemolytic episode in these patients.^14^ Although the majority of patients have asymptomatic G6PD deficiency or are unaware of this enzyme deficiency, exposure of red cells to oxidants such as drugs, ingestion of fava beans, and/or infection may result in acute hemolytic attack. In addition, it seems that this disease has no impact on longevity, abilities, and life quality of patients.^13^^, ^^15^The severity of hemolysis depends on different factors such as age, liver function, and concomitant use of drugs. The total bilirubin level can be increased by hepatitis as well as hemolysis that can lead to incorrect diagnosis when hepatitis causes hemolysis in these patients.^13^ Acute renal failure is also a potentially serious complication in acute viral hepatitis and concomitant G6PD deficiency. Acute renal failure is rare in children with G6PD deficiency.^13^ In a study in Nigeria, the prevalence of anemia in patients with acute viral hepatitis was reported as 24%.^7^ Moreover, severe anemia is not a common finding in acute viral hepatitis.^16^ Anemia in patients with viral hepatitis happens due to different reasons including transient suppression of bone marrow, autoimmune hemolytic anemia, dilutional anemia induced by the expansion of plasma volume, and reduced red blood cell (RBC) lifespan caused by extravascular defect.^7^^,^^9^^,^^17^^,^^19^ There are no studies regarding the prevalence of G6PD deficiency in patients with hepatitis A in Iran. However, severe hemolysis has been reported in hepatitis A, B, E, and concomitant G6PD deficiency.^20^^,^^21^Gotsman et al. investigated 200 patients with hepatitis A and found G6PD deficiency only in 18 patients (9%), of whom 44% had hemolysis. It was also found that patients with acute viral hepatitis and concomitant G6PD deficiency have significantly prolonged prothrombin time, higher temperature, elevated serum bilirubin level, and increased leukocyte count,^22^whereas in this study 26.3% of patients had G6PD deficiency, and there was no significant difference in the mean hemoglobin level between children with G6PD deficiency and children with normal G6PD level. Another study reported that two children who developed severe hemolysis during the course of hepatitis A had G6PD deficiency and autoimmune antibodies.^23^In this study, none of the patients developed hemolysis and renal dysfunction, or needed blood transfusion, moreover, there was no difference in hemoglobin level between patients with G6PD deficiency (7.2-14.8 gr/dl) and patients with normal G6PD level (7.6-15 gr/dl). Although the mean level of hemoglobin in patients with G6PD deficiency was lower than patients with normal G6PD level in this study, this difference was not statistically significant. 

## CONCLUSION

 Precise monitoring of patients with hepatitis A for symptoms of hemolysis and renal dysfunction is still recommended in regions with high prevalence of G6PD deficiency. It is also recommended to measure G6PD level of hepatitis A patients who are unaware of their G6PD level in malarious areas and regions with highly prevalent G6PD deficiency. Meanwhile, these patients are recommended to avoid taking medication and oxidizing agents.
